# Influence of *CYP19A1* polymorphisms on the treatment of breast cancer with aromatase inhibitors: a systematic review and meta-analysis

**DOI:** 10.1186/s12916-015-0373-9

**Published:** 2015-06-11

**Authors:** Osvaldo Artigalás, Tazio Vanni, Mara Helena Hutz, Patricia Ashton-Prolla, Ida Vanessa Schwartz

**Affiliations:** Postgraduate Program in Genetics and Molecular Biology, Department of Genetics, UFRGS, Av. Bento Gonçalves, 9500 - Prédio 43323M CEP: 91501-970 - Caixa Postal 15053 Porto Alegre, Rio Grande do Sul Brazil; Genetics Unit, Children’s Hospital, Grupo Hospitalar Conceição, GHC, Av. Francisco Trein, 596, CEP 91350-200 Porto Alegre, RS Brazil; Coordenação Geral de Avaliação de Tecnologias em Saúde – CGATS, Department of Science and Technology, Ministry of Health, SCN Quadra 02 Projeção C Subsolo Sala T-004, CEP: 70712-902 Brasília, DF Brazil; Medical Genetics Service, Hospital de Clinicas de Porto Alegre, HCPA, Rua Ramiro Barcelos, 2350, CEP: 90035-903 Porto Alegre, RS Brazil

**Keywords:** Adverse effects, Aromatase inhibitors, Breast cancer, Clinical outcomes, *CYP19A1*, Meta-analysis, Pharmacogenetics, Systematic review

## Abstract

**Background:**

Many clinical trials have shown the efficacy of aromatase inhibitors (AIs) in the management of breast cancer (BC). There is growing evidence that *CYP19A1* single-nucleotide polymorphisms (SNPs) are associated with clinical response (CR) and adverse effects (AEs) among BC patients treated with AIs. The aim of this study was to analyze the association between *CYP19A1* polymorphisms and AI treatment in BC patients.

**Methods:**

A systematic review was performed in MEDLINE, EMBASE, and LILACS. A meta-analysis was conducted to compare the association between *CYP19A1* variants and treatment response among BC patients.

**Results:**

A total of 12 studies were included in the final analysis. There was significant variation among the populations studied and the SNPs and outcomes investigated. A meta-analysis was only possible for the evaluation of SNP rs4646 vs. the wild-type variant with respect to time to progression (TTP) among metastatic BC patients treated with AI. TTP was significantly increased in patients with the rs4646 variant compared with the wild-type gene (hazard ratio (HR) = 0.51 [95 % confidence interval (CI), 0.33–0.78], *P* = 0.002). Seven studies analyzed the association between AEs with different polymorphisms of *CYP19A1*. Although there was a statistically significant association with musculoskeletal adverse events (rs934635, rs60271534, rs700518rs, and haplotype M_3_5) and with vasomotor symptoms (rs934635, rs1694189, rs7176005, and haplotype M_5_3) in individual studies, similar associations were not observed in further studies. No statistically significant association between musculoskeletal AEs and SNPs rs4646, rs10046, rs727479, and rs1062033 was found.

**Conclusions:**

These findings suggest that the presence of the rs4646 variant may be a predictive factor of the benefit of AI treatment for BC. The effects of *CYP19A1* polymorphisms on clinical outcomes were most often detected in individual studies, suggesting that longer-term studies will better clarify these associations. Additional studies are needed to clarify the predictive value of other SNPs and whether *CYP19A1* genotyping should be used to guide AI treatment.

## Background

Breast cancer (BC) is the most common cancer among women, and it accounts for the majority of cancer-related deaths among women worldwide, representing 23 % of all cancer diagnoses and 14 % of cancer-related deaths. In developing countries, BC has also replaced cervical cancer as the current leading cause of cancer deaths among women [1, 2].

Several studies have identified the role of estrogen and its metabolites in the development of BC [3, 4]. Initially, tamoxifen (a selective estrogen receptor modulator) was considered to be the optimal treatment for hormone-responsive BC in both premenopausal and postmenopausal women [5]. Recently, aromatase inhibitors (AIs) have emerged as relatively novel therapeutic options for BC patients [6].

AIs are classified as steroidal (type I, exemestane) and nonsteroidal (type II, anastrozole and letrozole) [7]. Currently, three AIs have been approved by the US FDA and the European Medicines Agency (EMA) for use in postmenopausal women with hormone receptor-positive BC at both the adjuvant and metastatic stages [8]. AI treatment improves disease-free survival (DFS), and lowers the rates of local recurrence, metastatic recurrence, and the incidence of contralateral BC compared with tamoxifen when used as an adjuvant therapy in postmenopausal women with estrogen receptor-positive (ER+) BC [9, 10]. AIs produce significantly lower recurrence rates compared with tamoxifen, either as initial monotherapy or after 2 to 3 years of tamoxifen [9, 10].

Aromatase is a cytochrome P450 enzyme complex that is encoded by *CYP19* located on chromosome 15q21.2 [11] and that catalyzes a critical reaction in estrogen biosynthesis involving the formation of aromatic C18 estrogens (estrone and estradiol) from C19 androgens (androstenedione and testosterone) [12]. It is expressed especially in the ovaries as well as several extragonadal tissues (subcutaneous fat, brain, liver, bone, vascular endothelial tissues, and the mesenchymal cells of the adipose tissue in the breast) [12]. Ma et al. [13] ‘resequenced’ all coding exons, all upstream untranslated exons plus their presumed core promoter regions, all exon-intron splice junctions, and a portion of the 3'-untranslated region of CYP19 using 240 DNA samples from patients of four ethnic groups and identified eighty-eight polymorphisms that resulted in 44 haplotypes. Many studies have reported an association between BC risk and the *CYP19A1* genotype [14–16].

Recently, following the publication of randomized clinical trials demonstrating the efficacy of AIs in the treatment of BC [17–19], additional studies have reported associations between *CYP19A1* polymorphisms and clinical response (CR) and/or adverse effects (AEs) in BC patients treated with AIs [20, 21]. Considering the potential associations of *CYP19A1* polymorphisms with BC risk, estrogen levels and variable aromatase activity levels, it is reasonable to propose that *CYP19A1* genotype has an impact on AI treatment response and ultimately patient survival [22].

Retrospective cohort and case–control studies have been published assessing the role of *CYP19A1* variants; however, most of these studies involved patients with different characteristics and included only small sample sizes. Consequently, their results are subjected to considerable heterogeneity and uncertainty. The present study is the first to systematically review the international literature and to conduct a meta-analysis of current studies to assess the associations of the *CYP19A1* genotype with clinical outcomes and AEs in BC patients treated with AIs.

## Methods

This meta-analysis was performed and reported according to the Preferred Reporting Items for Systematic Reviews and Meta-Analyses (PRISMA) guidelines [23].

### Search strategy

MEDLINE, EMBASE, LILACS, and Cochrane databases were searched using the following terms: (“aromatase inhibitor” OR “anastrozole” OR “letrozole” OR “exemestane”) AND (“cyp19a1” OR “aromatase gene” OR “aromatase polymorphism” OR “human cytochrome p450 aromatase” OR “cytochrome p450 19a1” OR “cyp19”). The search was performed on March 30, 2015. No language restrictions were applied. All references from review articles and retrieved articles were screened for additional publications on the subject. A second search was conducted in MEDLINE using the terms “aromatase inhibitors” AND “breast cancer” with the following filters: “clinical trial” AND “published in the last 10 years” AND “English”. A third search was performed using the following strategy: (“first and last author of the articles from the previous search”) AND (“cyp19a1” OR “aromatase gene” OR “aromatase polymorphism” OR “human cytochrome p450 aromatase” OR “cytochrome p450 19a1” OR “CYP19”) OR (“pharmacogenomics” OR “pharmacogenetics”). All titles and abstracts were screened by two independent researchers (OA and TV). During the data extraction process, complementary information not available in the selected articles was also searched for in clinical trial registries (http://www.clinicaltrials.gov, http://www.controlledtrials.com, and the Cochrane Register of Controlled Trials); we also contacted the authors of an included article [24] seeking specific details, but our efforts were unsuccessful.

### Selection criteria

Studies met the inclusion criteria and were considered eligible if they involved women with BC who were treated with AIs (letrozole, anastrozole, or exemestane), genotyped for *CYP19A1* and if an assessment of clinical outcomes was included. Non-clinical outcomes or pharmacodynamic studies, case reports, reviews and opinions as well studies of AIs other than letrozole, anastrozole, or exemestane were excluded. If multiple article from the same study were identified, the most recent publication was included in the analysis.

### Assessments and data extraction

Two investigators (OA and TV) independently reviewed, evaluated and extracted data from each included article. Conflicting evaluations were resolved following a discussion with a third reviewer (IVS). The review was performed according to the Cochrane Collaboration guidelines [25, 26].

Both authors independently extracted information using pre-designed forms. The following information was extracted: study design, characteristics of participants, setting, intervention, treatment duration, clinical outcome, and AEs. When studies included several subgroups that did not fulfill the inclusion criteria, only those subgroups that met the inclusion criteria were included.

### Statistical analysis

Summary hazard ratios (HRs) and corresponding 95 % confidence intervals (CIs) were estimated for time to progression (TTP). Statistical heterogeneity was assessed by performing the *χ*^2^ test (assessing the *P* value) and by calculating the *I*^2^ statistic. If *P* <0.10 and *I*^2^ > 50 %, indicating heterogeneity, a random-effects model was used; otherwise, the fixed-effects model was used [27]. Analyses were performed using the “*metan*” package in STATA version 13.0 (StataCorp LP, College Station, Texas, USA) [27]. This package uses the Mantel-Haenszel method to calculate the fixed-effects model. A Forest plot was used to illustrate the results of the meta-analysis.

## Results

### Search results

**Fig. 1:**
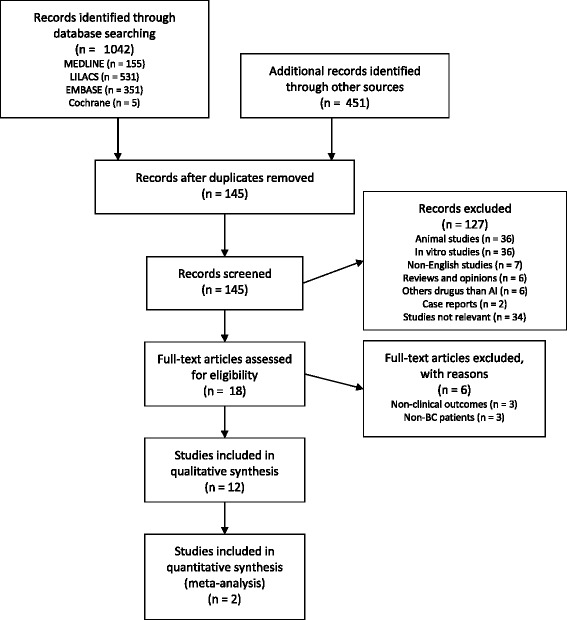
Flow diagram for article selection in the meta-analysis/systematic review

The literature search identified 145 publications subject to revision (Fig. 1). A total of 127 articles were rejected after the titles and abstracts were screened, since these were animal models, in vitro studies, or involved drugs other than AIs. Non-English studies and reviews or expert opinions were also rejected. Eighteen articles were fully evaluated. Six were excluded because they did not report relevant clinical outcomes (hormonal and gene expression levels) [24, 28, 29] or assessed AIs in non-BC patients (endometrial cancer) [30–32]. Therefore, 12 studies in total were included. Among these, three assessed AIs as neoadjuvant therapies [33–35], four assessed AIs as adjuvant therapies for patients with stages 0–III BC [36–39], and five assessed AIs as adjuvant therapies for patients with advanced breast cancer (ABC) [20, 21, 24, 40, 41] (Table 1).

**Table 1:** Characteristics and methodological aspects of studies included in the meta-analysis/systematic review

Study (year)	Country	Ethnicity	No. of patients	Clinical set	Menopausal status	Aromatase inhibitors	Main SNPs^a^/haplotypes (total analyzed)	Main outcomes^b^
Fontein et al. [39]	Netherlands	C	737	Adj	Post-menopausal	E	rs934635, rs1694189, rs7176005 (30)	AE (MS-AI, VMS)
Liu et al. [24]	China	As	272	ABC	NR	A	rs10046, rs4646 (2)	OS, TTP, AE
Miron et al. [40]	Italy	C	53	NR	NR	A, E, L	rs10046, rs4646, rs727479, rs700518 (4)	DFS, OS, TTP
Napoli et al. [38]	USA	C (80 %), B (19 %), As (1 %)	101	BC Stage 0-III	Post-menopausal	A, E, L	rs4646, rs700518, rs1062033 (3)	AE (MS-AI)
Ghimenti et al. [34]	Italy	C	37	Neoadj	Post-menopausal	A	rs7176005, rs6493497 (2)	CR (RECIST)
Ferraldeschi et al. [41]	UK	C (90 %), others (10 %)	308	ABC	NR	A, E, L	rs4775936, rs60271534, rs10459592, rs11636639, rs8039089 (56)	TTF
Park et al. [21]	Korea	As	109	ABC	Pre-/post-menopausal	L	rs700518, rs10459592, rs4775936 (46) * Haplotypes: M_1_3, M_2_1, M_3_5, M_5_3 (8)	CR (RECIST), TTP, AE (hot flushes), AE (MS-AI)
Mao et al. [37]	USA	C	390	Adjuvant BC Stage 0-III	Post-menopausal	A, E, L	rs749292, rs727479, rs10046, rs11575899, rs60271534 (5)	AE (MS-AI)
Garcia-Casado et al. [35]	Spain	C	95	Neoadj	Post-menopausal	L	rs10046, rs4646, rs700519 (3)	CR (WHO), PFS
Wang et al. [33]	USA, UK	C	45	Neoadj	NR	A, E, L	rs6493497, rs7176005 (48)	Tumor size
Colomer et al. [20]	Spain	C	67	ABC	Post-menopausal	L	rs10046, rs4646, rs727479 (3)	TTP, AE, CR (RECIST)
Henry et al. [36]	USA	C (88 %), B (9 %)	432	Adj	Post-menopausal	E, L	rs10046, rs1008805, rs10459592, rs1062033, rs11575899, rs2008691, rs2289105, rs2414096, rs28566535, rs28757184, rs3759811, rs4646, rs4774585, rs4775936, rs6493497, rs700518, rs7181886, rs727479, rs730154, rs749292, rs752760, rs936308, rs60271534 (24*)	AE (MS-AI), treatment discontinuation

*Abbreviations:* A, Anastrozole; ABC, Advanced breast cancer; AE, Adverse effect; As, Asian; B, Black; BC Stage 0-III, Breast Cancer stage 0-IIIA; C, Caucasian; CR (RECIST), Clinical response by RECIST criteria; CR (WHO), Clinical response by World Health Organization criteria; DFS, Disease-free survival; E, Exemestane; L, Letrozole; MS-AI, Musculoskeletal symptoms related to aromatase inhibitors; NR, Not reported; OS, Overall survival; PFS, Progression-free survival; SNPs, Single-nucleotide polymorphisms; TTF, Time to treatment failure; TTP, Time to progression; UK, United Kingdom; USA, United States of America; VMS, Vasomotor symptoms

^a^ Main SNPs with statistically significant results discussed and analyzed in this study

^b^ Main outcomes selected for analysis in this study

### Characteristics of the included studies

Most studies (n = 10) involved Caucasian patients. Two included African Americans, who represented less than 10 % of the total sample, another two included Asians, who represented less than 20 % of the total sample. Seven studies included only postmenopausal patients [20, 34–39]. One study included both pre- and postmenopausal patients [21], and four studies did not clearly state this information. In three studies, the sample populations consisted only of patients who received letrozole [20, 21, 35], in two studies, patients received anastrozole [24, 34], and in one study, patients received exemestane alone [39]. In the six remaining studies, the sample populations were composed of women who received any of the three AIs (anastrozole, letrozole, or exemestane).

Association analyses between outcome and *CYP19* polymorphisms were heterogeneous. Four studies examined TTP [20, 21, 24, 40], and only two examined overall survival (OS) [24, 40]. One study examined DFS [38], one examined time to treatment failure (TTF) [41], and one examined progression-free survival (PFS) [35]. In addition, Park et al. [22] correlated *CYP19* polymorphisms with the clinical benefits (CBs) of AIs according to Response Evaluation Criteria In Solid Tumors (RECIST), version 1.0. The AEs of AI were described in seven studies [20, 21, 24, 36–39].

**Fig. 2:**
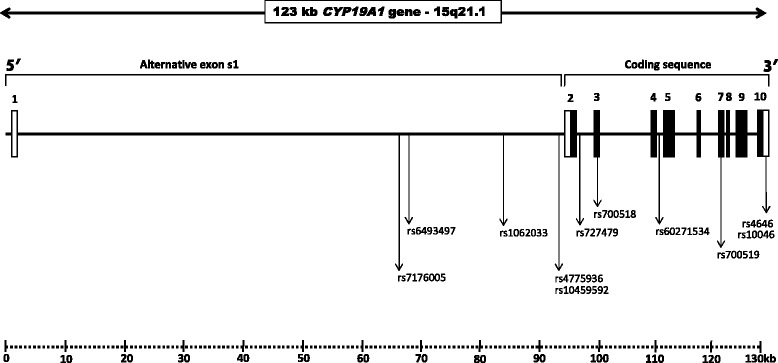
Genomic organization of *CYP19A1* gene and the location of key SNPs. Nine coding exons are indicated in black boxes and non-coding regions indicated by white boxes. Position number refers to the +1ATG start codon of GeneBank accession number NC_000015

In Fig. 2, we describe the key single nucleotide polymorphisms (SNPs) analyzed below, describing their genomic location within the *CYP19* gene.

### TTP and *CYP19A1* variants

**Fig. 3:**
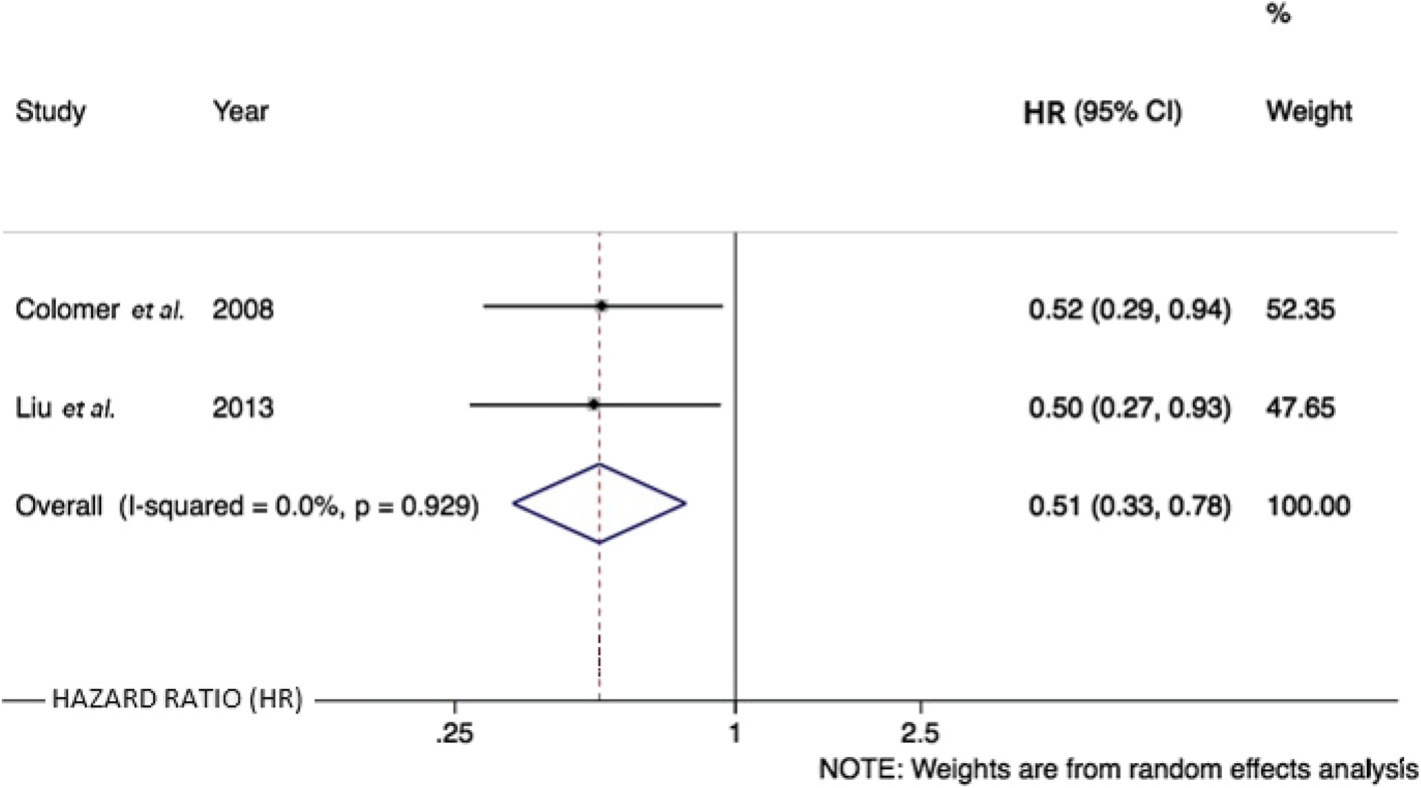
Forest plot showing the association of SNP rs4646 with TTP

Four studies reported an association between *CYP19A1* polymorphisms and TTP in women with metastatic ABC [20, 21, 24, 40]. The SNP rs4646 was analyzed in two studies [20, 24], which were meta-analyzed. This analysis demonstrated that TTP is significantly increased in patients with the rs4646 T allele (in homo- or heterozygosis) compared with patients with only wild-type alleles (hazard ratio (HR) = 0.51 [95 % CI, 0.33–0.78], *P* = 0.002; Fig. 3). No statistical heterogeneity was detected in these analyses (*χ*^2^ = 0.01, *P* = 0.929; *I*^2^ = 0.0 %).

Colomer et al. [20] also analyzed the associations of TTP in relation to two other SNPs but this relationship was not observed for the rs10046 variant (288 vs. 500 days; *P* = 0.3) or for the rs727479 variant (370 vs. 294 days; *P* = 0.9). Liu et al. [24] reported the absence of an association between rs10046 variants and TTP (14.93 months vs. 16.89 months; *P* = 0.94). Miron et al. [40] did not identify associations between TTP and SNPs, rs10046 (*P* = 0.070), and rs727479 (*P* = 0.052). However, they observed an association between increased TTP and the presence of the rs700518 G allele (*P* = 0.035).

Park et al. [21] performed a haplotype analysis and reported that M_1_3 is associated with a significantly longer TTP (11.08 months [95 % CI, 6.75–15.42] vs. 5.61 months in non-M_1_3 patients [95 % CI, 0.00–11.45], *P* = 0.040) in addition to M_2_1 (12.95 months [95 % CI, 9.27–16.63] vs. 7.31 months in non-M_2_1 patients [95 % CI, 4.63–9.99], *P* = 0.038). When the analysis considered only the individual variants (in a two-way analysis: homozygous vs. heterozygous), no significant difference in median TTP was observed between SNP carriers and wild-type individuals for rs700518 (12.07 months [95 % CI, 8.67–15.46] vs. 7.54 months [95 % CI, 6.53–8.55], *P* = 0.097), rs4775936 (11.93 months [95 % CI, 8.83–15.04] vs. 7.54 months [95 % CI, 6.57–8.51], *P* = 0.205) or rs10459592 (11.93 months [95 % CI, 8.66–15.21] vs. 7.74 months [95 % CI, 6.51–8.97], *P* = 0.176).

### OS and *CYP19A1* variants

The association between rs4646 and OS was described in two studies. Liu et al. [24] reported a statistically significant association between the presence of one or two rs4646 T alleles (G/T or T/T) and increased OS in women with metastatic BC: 37.3 months vs. 31.6 months (HR, 2.37 [95 % CI, 1.20–4.65], *P* = 0.001). However, Miron et al. [40] did not detect a significant association with OS after analyzing the same SNP. SNP rs10046 was also studied in these two papers. Miron et al. [40] described a significantly increased OS in patients carrying the T allele (*P* = 0.046). However, Liu et al. [24] failed to observe a similar association with OS (*P* >0.050). In addition, Miron et al. [40] studied two other SNPs, including rs727479, which was also associated with significantly increased OS when heterozygous (T/G) (*P* = 0.003) or homozygous (T/T) (*P* = 0.080). In the same study, rs700518 was not associated with a change in OS (*P* >0.050).

### DFS and *CYP19A1* variants

Miron et al. [40] performed the only study examining DFS, which included 53 patients and assessed four SNPs. The presence of SNPs rs4646 (*P* >0.050), rs10046 (*P* = 0.630), or rs700518 (*P* >0.050) was not associated with DFS. The presence of SNP rs727479 T allele was associated with increased DFS in both heterozygous (G/T) (*P* = 0.011) and homozygous (T/T) individuals (*P* = 0.040).

### TTF and *CYP19A1* variants

Ferradelschi et al. [41] examined TTF in 308 women with metastatic BC treated with AIs, including 56 variants of *CYP19A1*. Patients carrying the rs4775936 T allele were found to exhibit significantly increased TTF relative to patients with the reference allele (HR, 0.79 [95 % CI, 0.66–0.95], *P* = 0.012). Similar results were observed in patients with more than 7 TTTA repeats in SNP rs60271534 compared with fewer repeats (HR, 0.84 [95 % CI, 0.7–0.99], *P* = 0.04). Notably, when subjected to a multivariate analysis, these SNPs exhibited no significant association with TTF.

### PFS and *CYP19A1* variants

Garcia-Casado et al. [35] examined PFS in 95 women with postmenopausal BC treated with neoadjuvant letrozole with a median follow-up time of 40.6 months. Patients with the rs4646 A allele [in either heterozygous (A/C) or homozygous (A/A) status] did not present a significant reduction in PFS (85.7 % vs. 50.9 %; *P* = 0.0686). However, in a subgroup analysis of women who were not subjected to surgery after letrozole induction, this association was significant (100 % vs. 44.1 %; *P* = 0.009).

### CR and *CYP19A1* variants

CRs were assessed by measuring the size of lesions either by direct comparisons of tumor size [32] or using the RECIST score [21, 34].

Wang et al. [33] described the absence of any association (*P* >0.05) between 48 SNPs (including rs6493497 and rs7176005) and tumor size in pre- and post-AI neoadjuvant treatment in 52 women with BC. Considering responsiveness to treatment as a ≥30 % reduction in tumor volume, Ghimenti et al. [34] did not observe a statistically significant association in relation to SNPs rs6493497 and rs7176005 in a neoadjuvant scenario. Park et al. [21] allocated patients achieving a complete response or a partial response or patients with stable disease for more than 6 months (standard deviation of approximately 6 months) to the CB group. Patients presenting progressive disease or stable disease for <6 months during treatment were placed in the non-CB group. They analyzed 47 SNPs and identified statistically significant associations between CB and rs700518 (OR, 2.52 [95 % CI, 1.02–6.20]; *P* = 0.044), rs10459592 (OR, 2.61 [95 % CI, 1.6–6.46]; *P* = 0.038) and rs4775936 (OR, 2.89 [95 % CI, 1.16–7.22]; *P* = 0.023) after adjusting for age, HER2 positivity, number of metastatic lesions, and liver metastasis. Haplotype analysis revealed an association between CB and haplotypes M_1_3 (OR = 5.33 [95 % CI, 1.63–17.45]; *P* = 0.006) and M_2_1 (OR, 3.37 [95 % CI, 1.43–7.90]; *P* = 0005).

### AEs and *CYP19A1* variants

Compared with tamoxifen, AIs are associated with higher risks of osteoporosis, fractures, cardiovascular complications, and hypercholesterolemia. In addition, AIs are associated with musculoskeletal side effects and may block ovarian function causing, for example, hot flushes [42]. AEs were reported in seven of the 11 articles included in this review. Nonetheless, one of the seven articles only reported the presence or absence of any AE [20], and they did not observe significantly increased frequencies of any AEs when analyzing SNPs rs4646, rs10046, or rs727479 in 67 women with postmenopausal ABC treated with letrozole. No patient in this study interrupted treatment due to AEs.

### Vasomotor symptoms (VMS)

Fontein et al. [39] observed 737 patients receiving adjuvant exemestane and noted that the homozygous AA genotype of rs934635 was associated with a significantly higher odds of VMS (univariate analysis OR, 2.86 [95 % CI, 1.12–7.27]; *P* = 0.044, and multivariate analysis OR, 2.78 [95 % CI, 1.02–7.56]; *P* = 0.044). In addition, for rs7176005, the homozygous variant genotypes (TT) were associated with a higher odds of VMS (univariate OR, 6.36 [95 % CI, 1.5–27.0]; *P* = 0.021, and multivariate OR, 4.9 [95 % CI, 1.02–23.5]; *P* = 0.06). Finally, the rs16964189 SNP was associated with the occurrence of VMSs for the homozygous TT genotype (univariate OR, 1.76 [95 % CI, 0.79–3.92]; *P* = 0.025 and multivariate OR, 1.86 [95 % CI, 0.76–4.59]; *P* = 0.06). Park et al. [21] described an association between haplotype M_5_3 (including rs1902586, rs7181886, rs936306, rs1902582, rs16964254, and rs28566535) and hot flushes (OR, 4.12, [95 % CI, 1.09–15.61], *P* = 0.03).

### Musculoskeletal adverse events (MS-AEs)

In relation to treatment-related MS-AEs, Liu et al. [24] found that the proportion of AEs among women with metastatic BC treated with anastrozole did not differ when stratified by SNPs rs4646 (*P* = 0.894), rs10046, rs2830, rs9926298, and rs9939609 (data not shown). Fontein et al. [39] reported that the homozygous AA genotype of rs934635 was associated with a significantly higher odds of MS-AEs compared to wild-type GG and GA, with an OR, 4.62 [95 % CI, 1.79–12.0]; *P* = 0.007 in the univariate analysis. Multivariate analyses were adjusted for age, BMI, and adjuvant chemotherapy and revealed an OR, 5.08 [95 % CI, 1.8–14.3]; *P* = 0.007. However, this association was not found in any other of the 29 SNPs analyzed. Furthermore, Henry et al. [36] analyzed 138 variants in 24 genes (including 23 *CYP19A1* SNPs) in 432 BC patients and observed a non-significant increase in MS-AEs when at least eight rs60271534 repeat alleles were present (HR, 1.8 [95 % CI, 0.8–1.8], *P* = 0.49). No other associations were identified.

Park et al. [21] described the association between haplotype M_3_5 (including rs12148604, rs4646, rs10046, rs700519, rs4324076, rs700518, rs3759811, rs727479, rs4775936, rs10459592, rs767199, rs10519297, rs1062033, rs2008691, rs1008805, and rs17523527) and MS-AEs (bone pain and arthralgia) in 66 of 109 patients included in their study (OR, 11.25 [95 % CI, 1.17–108.28], *P* = 0.01), but no other significant associations were reported. Finally, Mao et al. [37] analyzed five SNPs in 390 patients, reporting no association between MS-AEs and SNPs rs749292 (*P* = 0.57), rs727479 (*P* = 0.94), rs10046 (*P* = 0.20), or rs11575899 (*P* = 0.80). For SNP rs60271534, subjects with at least one TTTA 7-repeat allele had a non-significant 1.7-fold increase in odds of AIAA (OR, 1.70 [95 % CI, 1.06–2.73]; *P* = 0.028) after correcting for multiple testing, whereas patients with at least one TTTA 8-repeat allele had a lower risk of aromatase inhibitor-associated arthralgia (AIAA) (OR, 0.41 [95 % CI, 0.21–0.79]; *P* = 0.008). In addition, Napoli et al. [38] analyzed 97 patients treated with AIs and assessed the possible associations between SNPs rs4646, rs700518, and rs1062033 and bone loss. They did not observe any significant phenotypic differences in patients with distinct genotypes with respect to rs4646 and rs1062033. The presence of the homozygous rs700518 A allele (AA) was associated with a greater loss of bone mineral density in both the lumbar spine (*P* = 0.03) and hip (*P* = 0.03) compared with other genotypes (AG + GG).

## Discussion

There is growing evidence that polymorphic gene variants can contribute to differences in complex traits between individuals, and the assessment of genes involved in drug metabolism will provide valuable information for treatment planning. Although AI efficacy among BC patients has been proven, there is significant variability in response rate and AE frequency. Therefore, tests that are able to predict treatment response and prognosis would be valuable in the management of these patients. We investigated evidence of the association between *CYP19A1* genotypes and clinical outcomes following treatment with AIs in BC patients. A total of 12 studies were included in this systematic review. To our knowledge, this is the first systematic review and meta-analysis published on the association between *CYP19A1* polymorphisms and AI treatment in BC patients.

TTP was analyzed as an outcome in four studies [20, 21, 24, 40]. In this meta-analysis, SNP rs4646 was the only SNP associated with increased TTP, suggesting that this variant is likely involved in the response to AI throughout tumoral evolution. SNPs rs10046 [20, 24, 40], rs700518 [21, 40], and rs727479 [20, 24] did not exhibit any associations with TTP. Due to the heterogeneity between the patient groups, pooling of data was only possible for SNP rs4646.

Liu et al. [24] reported an association between SNP rs4646 and increased OS (*P* = 0.007), but the same was not observed for SNP rs10046 (*P* >0.05). In contrast, Miron et al. [40] found no association between OS and rs4646 (*P* >0.05) but did identify an association between OS and rs10046 (*P* = 0.003). These conflicting data suggest that *CYP19A1* genotypes may be associated with OS in BC patients treated with AIs. However, the magnitude of this association appears highly variable between patients.

The variations in the definitions of DFS, PFS and TTF among studies limited pooling in our analysis. The results involving DFS (positive association with SNP rs727479, *P* = 0.011), TTF and PFS (no significance association with any SNP) were quite limited in terms of number and heterogeneity of studies, but the overall lack of effect observed may indicate that there are in fact no differences or that the follow-up times used were insufficient to detect relapse/recurrence.

Six studies reported AEs related to AIs [20, 21, 24, 36–38], but only five studies specifically described these AEs as hot flushes [21] or as musculoskeletal complaints [20, 24, 36–38], and the majority did not analyze their associations with SNPs. Notably, SNPs rs4646, rs10046, rs727479 and rs1062033 were evaluated in three studies. None of them reported significant associations between *CYP19A1* SNPs and MS-AEs arising from AI treatment [20, 24, 37, 38].

This systematic review and meta-analysis was subjected to limitations. There was inherent heterogeneity in the patient characteristics, polymorphisms, AIs used, clinical settings, and pretreatment regimens. Most of the studies included were retrospective. Therefore, we cannot exclude that other unknown confounders may have biased the results. Studies describing adherence to AI treatment are important because the limited data available have suggested that the most common reason for discontinuing treatment is MS-AEs, which was reported to be responsible for treatment discontinuation in 10–20 % of the patients in all of the included studies [43–45]. It is possible that reduced compliance by some patients may have led to underestimation of the benefits of AI treatment.

Furthermore, it is important to mention that this systematic review/meta-analysis might have some publication bias, because we prioritized the inclusion of English publications. Moreover, it is well known that often studies that find negative results, i.e., with no statistically significant correlation between genetic polymorphisms and clinical outcomes, are more rarely published, which may cause a possible limitation in this kind of study. This meta-analysis did not use single patient data, which although more challenging, may have certain advantages over aggregate-level analyses [46]. Furthermore, many of the works included herein were performed without knowledge of other polymorphisms (in *CYP19A1* gene, as well in other genes involved in steroidogenesis) that could influence the outcomes analyzed.

## Conclusions

This systematic review of the literature revealed associations between *CYP19A1* polymorphisms and clinical outcomes and AEs in BC patients receiving AIs. The effects of *CYP19A1* polymorphisms on clinical outcomes were most often detected in individual studies, suggesting that longer-term studies will better clarify these associations. Results, such as the association of SNP rs4646 with longer TTP as well as the association of rs934635 and M_5_3 haplotype with VMS and the genetic variants M_3_5, rs700518 and rs60271534 with osteoarticular symptoms, are significant outcomes indicating the impact of these variants on treatment with AIs in BC. Hence, *CYP19A1* polymorphisms are potentially useful biomarkers for predicting prognosis and AE profiles in BC patients and may become a promising tool to aid physicians in making therapeutic decisions in the future, although the exact role of *CYP19A1* has yet to be clarified when taking the different clinical settings and disease stagings into account. Additional studies must be conducted with larger sample sizes, more homogeneous patient populations (including clinical, demographic, ethnic and genetic aspects), and using standardized outcomes and genotyping strategies to allow for a comprehensive understanding of these associations.

## Abbreviations

ABC: Advanced breast cancer
AEs: Adverse effects
AIs: Aromatase inhibitors
BC: Breast cancer
CB: Clinical benefit
CR: Clinical responses
DFS: Disease-free survival
FDA: US Food and Drug Administration
HRs: Hazard ratios
MS-AEs: Aromatase inhibitor-related musculoskeletal adverse events
OR: Odds ratio
OS: Overall survival
PFS: Progression-free survival
RECIST: Response Evaluation Criteria In Solid Tumors
SNP: Single nucleotide polymorphism
TTF: Time to treatment failure
TTP: Time to progression
VMS: Vasomotor symptoms
